# Firearm-associated ocular injuries: analysis of national trauma data

**DOI:** 10.5935/0004-2749.20210055

**Published:** 2025-02-02

**Authors:** Timothy Truong, Catherine Hua He, David Michael Poulsen, Afshin Parsikia, Joyce Nanjinga Mbekeani

**Affiliations:** 1 Department of Medicine, Bronxcare Health System, Bronx, NY, USA; 2 Albert Einstein College of Medicine, Bronx, NY, USA; 3 Casey Eye Institute, Oregon Health & Science University, Portland, OR, USA; 4 Department of Surgery (Trauma), Jacobi Medical Center, Bronx, NY, USA; 5 Research Services, University of Pennsylvania, PA, USA; 6 Department of Ophthalmology & Visual Sciences, Montefiore Medical Center/Albert Einstein College of Medicine, Bronx, NY, USA; 7 Department of Surgery (Ophthalmology), Jacobi Medical Center, Bronx, NY, USA

**Keywords:** Eye injuries, Firearms, Database, demographic disparity, Traumatismos oculares, Ferimentos por armas de fogo, Banco de dados

## Abstract

**Purpose:**

The United States of America has the highest gun ownership rate of all high-income nations, and firearms have been identified as a leading cause of ocular trauma and visual impairment. The purpose of this study was to characterize firearm-associated ocular injury and identify at-risk groups.

**Methods:**

Patients admitted with firearm-associated ocular injury were identified from the National Trauma Data Bank (2008-2014) using the International Classification of Diseases, Ninth Revision, Clinical Modification diagnostic codes and E-codes for external causes. Statistical analysis was performed using the SPSS 24 software. Significance was set at p<0.05.

**Results:**

Of the 235,254 patients, 8,715 (3.7%) admitted with firearm-associated trauma had ocular injuries. Mean (standard deviation) age was 33.8 (16.9) years. Most were males (85.7%), White (46.6%), and from the South (42.9%). Black patients comprised 35% of cases. Common injuries were orbital fractures (38.6%) and open globe injuries (34.7%). Frequent locations of injury were at home (43.8%) and on the street (21.4%). Black patients had the highest risk of experiencing assault (odds ratio [OR]: 9.0; 95% confidence interval [CI]: 8.02-10.11; p<0.001) and street location of injury (OR: 3.05; 95% CI: 2.74-3.39; p<0.001), while White patients had the highest risk of selfinflicted injury (OR: 10.53; 95% CI: 9.39-11.81; p<0.001) and home location of injury (OR: 3.64; 95% CI: 3.33-3.98; p<0.001). There was a steadily increasing risk of self-inflicted injuries with age peaking in those >80 years (OR: 12.01; 95% CI: 7.49-19.23; p<0.001). Mean (standard deviation) Glasgow Coma Scale and injury severity scores were 10 (5.5) and 18.6 (13.0), respectively. Most injuries (53.1%) were classified as severe or very severe injury, 64.6% had traumatic brain injury, and mortality occurred in 16% of cases.

**Conclusion:**

Most firearm-associated ocular injuries occurred in young, male, White, and Southern patients. Blacks were disproportionally affected. Most firearm-associated ocular injuries were sightthreatening and associated with traumatic brain injury. The majority survived, with potential long-term disabilities. The demographic differences identified in this study may represent potential targets for prevention.

## Abstract (pt)

**Objetivo:**

Os Estados Unidos têm a maior taxa de posse de armas de fogo de todos os países de alta renda e essas armas foram identificados como uma das maiores causas de trauma ocular e deficiência visual. O objetivo deste estudo foi caracterizar as lesões oculares associadas a armas de fogo e identificar grupos de risco.

**Métodos:**

Foram identificados pacientes hospitalizados com lesões oculares associadas a armas de fogo no período de 2008 a 2014, a partir do Banco de Dados Nacional de Trauma (*National Trauma Data Bank*), usando os códigos de diagnósticos da CID9MC e códigos “E” para causas externas. A análise estatística foi efetuada usando o programa SPSS. O nível de significância considerado foi de p<0,05.

**Resultados:**

De um total de 235.254 pacientes hospitalizados com trauma associado a armas de fogo, 8.715 (3,7%) tinham lesões oculares. A média de idade foi de 33,8 (DP 16,9) anos. A maioria foi de homens (85,7%), brancos (46,6%) e da região Sul (42,9%); 35% dos pacientes eram negros. As lesões mais comuns foram fraturas de órbita (38,6%) e lesões de globo aberto (34,7%). Os locais mais frequentes foram a residência (43,8%) e a rua (21,4%). Pacientes negros tiveram maior probabilidade de sofrer agressões (RP=9,0, IC 95%=8,02-10,11; p<0,001) e da ocorrência ser na rua (RP=3,05, IC 95%=2,74-3,39; p<0,001), enquanto pacientes brancos tiveram maior probabilidade de lesões autoprovocadas (RP=10,53, IC 95%=9,39-11,81; p<0,001) e da ocorrência ser na residência (RP=3,64, IC 95%=3,33-3,98; p<0,001). A probabilidade de lesões autoprovocadas aumentou com a idade de forma consistente, atingindo o máximo em pacientes com mais de 80 anos (RP=12,01, IC 95%=7,49-19,23; p<0,001). A pontuação média na escala de coma de Glasgow foi 10 (DP 5,5) e na escala de severidade da lesão foi 18,6 (DP 13,0). A maioria das lesões (53,1%) foi classificada como severa ou muito severa. Dentre os pacientes, 64,6% tiveram lesão cerebral traumática e 16% evoluíram a óbito.

**Conclusão:**

A maior parte das lesões oculares relacionadas a armas de fogo ocorreu em pacientes jovens, do sexo masculino, brancos e sulistas. Negros foram afetados desproporcionalmente. A maior parte das lesões oculares relacionadas a armas de fogo apresentou riscos à visão e foi associada a lesões cerebrais traumáticas. A maioria dos pacientes sobreviveu, mas com potencial para invalidez no longo prazo. As diferenças demográficas identificadas podem ser potencialmente alvos de ações preventivas.

## INTRODUCTION

The United States of America (USA) has firearm-associated injuries that far outnumber those of other affluent nations. In a cross-sectional analysis of high-income countries, the USA was found to contribute 80% of all firearm-associated deaths^(1)^ with a crude rate of 11.1 per 100,000 individuals in 2015^(2)^. A retrospective survey of firearm-associated injuries (2006-2014) estimated that the combined financial burden of emergency room visits, hospitalization, and lost wages was $45.6 billion per year^(3)^. Ocular trauma is a leading cause of monocular blindness in the USA and second only to cataracts as the most frequent cause of visual impairment^(4)^. Firearm injuries are a leading cause of ocular trauma, often resulting in permanently impaired vision and blindness^(5-11)^.

Firearm injuries, especially those afflicting the face and head, are associated with significant morbidity and mortality^(10-13)^. Fahimi et al.^(13)^ found that, even when compared with victims of motor vehicle accidents and assault not related to gunshot wounds, patients who survived firearm violence had a five-fold higher hazard of death in their first year after discharge. In one of very few studies of firearm-associated ocular injury (FAOI), Chopra et al.^(9)^ found that 44% of patients from two New York City hospitals who survived firearm injury suffered long-term visual disability, highlighting the impact of firearms on vision. Research focusing on FAOI on a national scale is limited. Thus, we utilized a large national database to characterize FAOIs by describing the circumstances and spectrum of ocular injuries and identify at-risk demographic groups.

## METHODS

This retrospective analysis of patient records from the National Trauma Data Bank (NTDB) between 2008 and 2014 was approved by the Institutional Review Board at the Montefiore Medical Center/Albert Einstein College of Medicine (Bronx, NY, USA). The NTDB, an American College of Surgeons-maintained database, aggregates de-identified patient data from >900 trauma centers to form one of the world’s largest trauma registries. These data provide nationally representative estimates of hospitalized patients with trauma.

### Subjects and methodology

We included all patients who were admitted, expired upon arrival, or expired after an initial evaluation who had International Classification of Diseases, Ninth Revision, Clinical Modification (ICD-9-CM) diagnosis codes of 800.00-959.9. Patients who had ocular trauma resulting from a firearm mechanism were identified. Ocular injuries included all sub-categories of superficial injury of the eye and adnexa (918.0-918.2, 918.9), burn to the eye and adnexa (940.0-940.5, 940.9), contusion of the eye and adnexa (921.0-921.4, 921.9, 364.0-364.1, 364.3), foreign body on the ocular surface (930.0-930.2, 930.8-930.9), foreign body inside the eye (871.5-871.6, 360.59-360.69), orbital injuries (802.6-802.9, 376.32376.33), open wound of the eyeball (871.0-871.7, 871.9), open wound of the ocular adnexa (870.0-870.9), optic nerve injury and/or visual pathways (950.0-950.3, 950.9), and cranial nerve injury other than optic nerve (951.0-951.4, 951.9). For other cranial nerves, we focused on those commonly observed in neuro-ophthalmic injuries comprising oculomotor, trochlear, abducens, trigeminal, and facial nerves. All types of firearms, including handguns, automatic shotguns, hunting rifles, and military firearms, for all intentions were identified using ICD-9 E-codes: unintentional (922.0-922.9), self-inflicted (955.0-955.9), assault (965.0-965.4), undetermined intent (985.0-985.4), and legal intervention (970.0).

From the selected patients, we documented demographic data, including age, gender, race and ethnicity, type of injuries, location, intent of injury, length of hospital stay, medical insurance, disposition upon discharge, trauma center designation level (I-IV), and USA geographic census region (Northeast, South, Midwest, West). Emergency department-determined Injury Severity Score (ISS) and Glasgow Coma Scale (GCS) were documented and used as indices of injury severity. ISS (1-75) is a scoring system that designates severity with increasing scores based on the degree and anatomical site of injury. GCS (0-15) is a common measure of the level of consciousness; low scores are assigned to greater traumatic brain injury (TBI). ISS ≥15 is designated major trauma and GCS ≤8 is considered severe TBI. The Center for Disease Control criteria were used to guide the identification of patients with TBI, using ICD-9-CM codes for skull fracture (800.0-801.9, 803.0-804.9), injury to the optic chiasm, optic pathway or visual cortex (950.1950.3), intracranial injury (850.0-854.1), and head injury not otherwise specified (959.01). The mortality rate was determined by assessing the types of discharge and included deaths on arrival and after admission.

### Statistical analysis

Analysis was conducted using the SPSS software (Statistical Package for Social Science, version 24; IBM Corp., Armonk, NY, USA). For all continuous variables, mean, standard deviation, median, and interquartile range values were calculated. For the logistic regression analysis, ages were stratified into decade groups. Similarly, ISS and GCS were grouped according to NTDB sub-classifications. For ISS, the grouping was as follows: minor (ISS: 1-8), moderate (ISS: 9-15), severe (ISS: 16-24), and very severe (ISS >24) injury. For GCS, the grouping was as follows: mild (GCS: 13-15), moderate (GCS: 9-12), and severe (GCS ≤8) brain injury. Associations between variables were analyzed using the paired Student’s t-test, chi-squared test, and logistic regression analysis. Charts and tables were generated using Microsoft Excel (Microsoft Corp, Redmond, WA, USA). Data points classified under “undetermined,” “not applicable”, or “unknown” were excluded from comparative analyses.

## RESULTS

**Figure 1:**
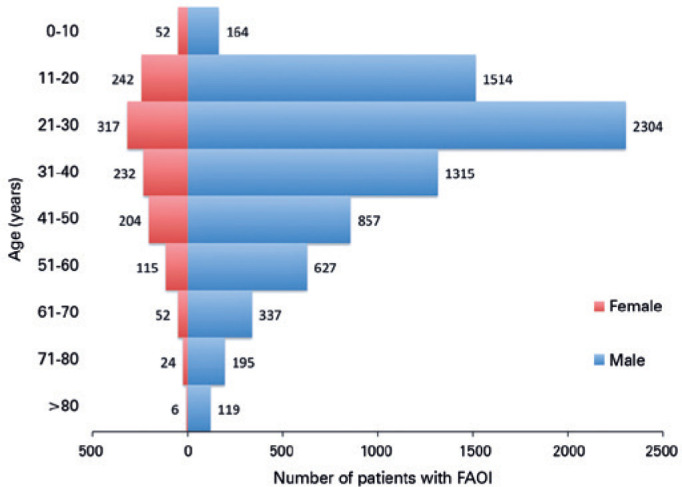
Age distribution of patients with firearm-associated ocular injury by age and gender, NTDB (2008-2014). Mean (SD) age of patients was 23.8 (16.9) years (range: 0-110 years). For both genders, >50% of patients were aged 11-40 years.

A total of 235,254 patients with firearm-associated trauma were admitted between 2008 and 2014, and 8,715 (3.7%) of those resulted in FAOI. This represented 2.75% of all ocular injuries (316,485) during this time period. When stratified by year of admission, the frequency of injuries was relatively stable, with an average of 1,245 per year (range: 1,173-1,351). The mean (standard deviation [SD]) age was 33.8 (16.9) years, with 22.6%, 70%, and 6.2% of cases classified in the pediatric (≤20 years), adult (21-65 years), and elderly (≥65 years) age groups, respectively. Males had similar mean (SD) age to women: 33.8 (17) and 33.7 (16) years, respectively. However, they had higher overall rates of FAOI (85.7% vs. 14.3%, respectively). In all age groups, males outnumbered women (Figure 1). Of all cases, Whites represented 46.6%, Blacks, 35%, and all other races, 18.4%. Hispanic ethnicity comprised 13.7%. Common locations of injury were at home (43.8%) and on the street (21.4%). Most cases were from the Southern (42.9%) and Northeast (21.5%) regions (Table 1).

**Table 1:** Descriptive findings and demographic data of firearm-associated ocular injuries, National Trauma Data Bank, 2008-2014

Characteristic	Number	%	Characteristic	Number	%	Mean (SD)	Median (1QR)
Year			Age (years)			23.8 (16.9)	29 (21-44)
2008	1,173	13.5	0-10	216	2.5		
2009	1,244	14.3	11-20	1,756	20.1		
2010	1,198	13.7	21-30	2,621	30.1		
2011	1,178	13.5	31-40	1,547	17.8		
2012	1,274	14.6	41-50	1,061	12.2		
2013	1,297	14.9	51-60	742	8.5		
2014	1,351	15.5	61-70	389	4.5		
Total	8,715	100.0	71-80	219	2.5		
			>80	125	1.4		
Gender							
Male	7,469	85.7					
Female	1,246	14.3	Hospital stay (days)			9.8 (14.6)	4(1-13)
			1	2,450	28.1		
Race			2-3	1,639	18.8		
Black	3,050	35.0	4-6	1,178	13.5		
White	4,065	46.6	>6	3,416	39.2		
Other	1,600	18.4	Unknown	32	0.4		
Hispanic	1,193	13.7					
			1SS			18.6 (13.0)	18 (9-26)
Hospital			1-8	2,026	23.2		
Level 1	3,915	44.9	-9-15	1,639	18.8		
Level 11	1,378	15.8	16-24	1,685	19.3		
Level 111	68	0.8	>24	2,948	33.8		
Level IV	14	0.2					
Not applicable	3,340	38.3					
			GCS			10.4 (5.3)	14 (3-15)
Locations			<8	3,321	38.1		
Home	3,815	43.8	9-12	418	4.8		
Street	1,868	21.4	13-15	4,347	49.9		
Public building	376	4.3	Unknown	629	7.2		
Recreation	153	1.8					
Residential Institution	23	0.3					
Industry							
Farm	17	0.2	TB1	5,634	64.6		
Other	719	8.2					
Unspecified	1,333	15.3	Mortality	1,409	16.2		
Unknown	370	4.3					
US regions			Intention				
Midwest	1,872	21.5	Assault	4,954	56.8		
Northeast	1,142	13.1	Self-inflicted	2,619	30.1		
South	3,742	42.9	Unintentional	808	9.3		
West	1,805	20.7	Other	1	0.0		
Not applicable	31	0.4	Undetermined	333	3.8		
Unknown	123	1.4	Unknown	0	0.0		

SD= standard deviation; IQR= interquartile range; ISS= injury severity score; GCS= Glasgow Coma Scale; TBI= traumatic brain injury.

SD= standard deviation; NTDB= National Trauma Data Bank.

Orbital injuries (28.6%), open globe wounds (34.7%), and contusions to the globe/adnexa (15.7%) were the most common FAOI. Associated TBI occurred in 64.6% of cases. Visual pathway injuries occurred in 7.92% of the patients. The optic nerve was most frequently affected (87.7%). The mean (SD) GCS score was 10 (5.5), and 38.1% of injuries were classified as severe brain injury (GCS ≤8). Similarly, the mean (SD) ISS was 18.6 (13.0), and 33.8% were classified as very severe injuries (ISS >24). Intention of injuries were assault (56.8%), self-inflicted (30.1%), and unintentional (9.3%). Most patients (48.8%) were discharged home and fewer patients (20.5%) were transferred to another facility. Mean (SD) hospital stay was 9.8 (14.6) days and the mortality rate was 16.2% (Table 1).

### Comparative analyses

#### Age and gender differences

Across all age groups, injuries occurred most frequently at home, with the highest risk observed in the two extreme age groups. Patients aged 0-10 years had a 2.87-fold higher risk (95% confidence interval [CI]: 2.153.84; p<0.001) of injury at home than elsewhere; those aged >80 years had the highest risk of injury at home (odds ratio [OR]: 5.84; 95% CI: 3.71-9.91; p<0.001) compared with other locations. The street was the most likely site in those aged 21-30 years (OR: 1.56; 95% CI: 1.49-1.74; p<0.001). The 11-20-year group had the highest risk of firearm injury in a recreational facility than other locations (OR: 1.61; 95% CI: 1.13-2.28; p=0.008), followed closely by the street (OR: 1.44; 95% CI: 1.28-1.63; p<0.001).

**Figure 2:**
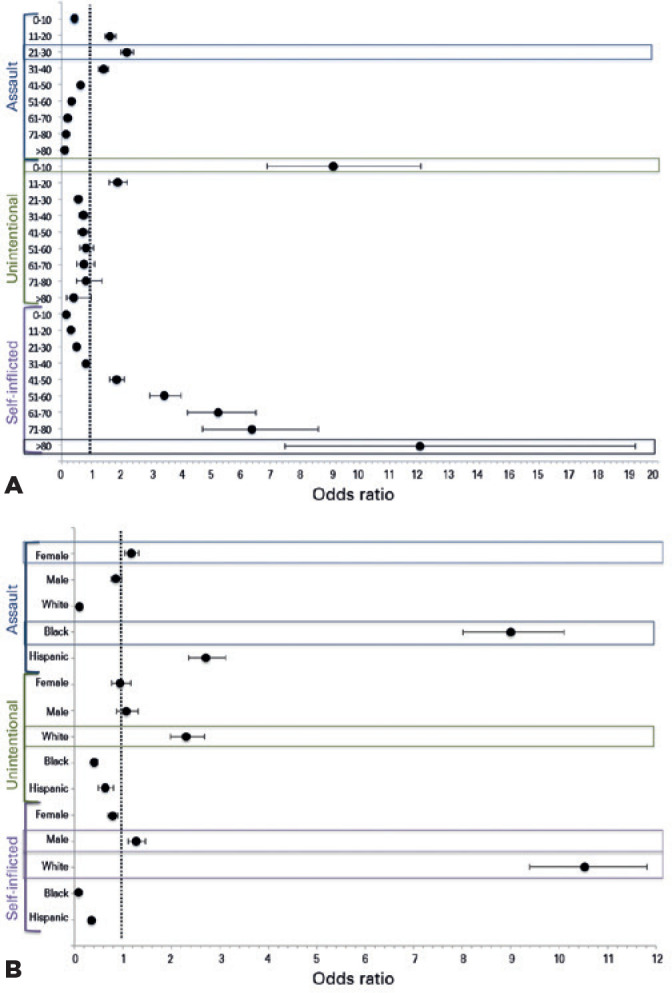
A) Simple logistic regression of intent of injury and age in patients with firearm-associated ocular injuries, NTDB (2008-2014). Summary of simple logistic regression with odds ratio and 95% confidence intervals analysis of intent of injury amongst different age groups with firearmassociated ocular injuries. Unintentional injury showed a strong association with the youngest age strata, 0-10 years with a 9-fold higher risk; p<0.001. including 9.1 odds of association with 0-10 years. Self-inflicted was most associated with the oldest age strata, >80 years with 12 -fold higher risk of association; p<0.001. Assault showed a strong association with strata between -20-30 years with a 2-fold higher risk of association; p<0.001. Boxed plots represent categories with the highest odds ratio. B) Simple logistic regression of intent of injury and gender, race, and ethnicity in firearm-associated ocular injuries, NTDB (2008-2014). Summary of simple logistic regression with odds ratio and 95% confidence intervals analysis of intent of injury and gender, race, and ethnicity in firearm-associated ocular injury. Assault was associated with female gender (OR: 1.2; p<0.001), and 9.0-fold and 2.7-fold odds of association with Blacks (p<0.001) and Hispanics (p<0.001), respectively. Unintentional injury had 2.3-fold odds of being associated with Whites (p<0.001) without significant gender association. Self-inflicted injury had an associated 10.5-fold odds of association with Whites (p<0.001) and 1.2-fold odds with males (p<0.001). Boxed plots represent categories with the highest odds ratio.

Patients aged between 0-10 years had a 9.11-fold higher risk (95% CI: 6.89-12.04; p<0.001) of sustaining FAOI due to unintentional injury. Meanwhile, teens and young adults had an increased risk of injury due to assault, with the 21-30-year group having the highest risk (OR: 2.19; 95% CI: 1.99-2.42; p<0.001). There was steadily increasing risk of self-inflicted injury from the 41-50 year group (OR: 1.85; 95% CI: 1.62-2.11; p<0.001) to a peak in the >80 year group (OR: 12.01; 95% CI: 7.4919.23; p<0.001) (Figure 2A). Males were at a higher risk of self-inflicted injury (OR: 1.27; 95% CI: 1.11-1.46; p<0.001) than other intentions. Males were also at a higher risk of injuries occurring in a recreational facility (OR: 1.81; 95% CI: 1.02-3.21; p=0.039), while females had the highest risk of assault (OR: 1.17; 95% CI: 1.06-1.33; p=0.01) and a higher likelihood of injury at home (OR: 1.63; 95% CI: 1.45-1.84; p<0.001) than other locations.

NTDB= National Trauma Data Bank; SD= standard deviation; OR= odds ratio.

### Race and ethnic differences

Hispanics and Blacks suffering injuries were more likely to be in the 11-20-year group (OR: 1.62; 95% CI: 1.41-1.86; p<0.001) and 21-30-year group (OR: 1.93; 95% CI: 1.76-2.12; p<0.001), respectively. On the other hand, Whites were the most elderly, with the highest risk of injury noted in the 71-80-year group (OR: 8.5; 95% CI: 5.67-12.75; p<0.001). Whites were at a higher risk of injury at home than other common locations (OR: 3.64; 95% CI: 3.33-3.98; p<0.001), while Blacks (OR: 3.05; 95% CI: 2.75-3.39; p<0.001) and Hispanics (OR: 1.62; 95% CI: 1.41-1.86; p<0.001) were at a higher risk of injury on the street.

With respect to intention, Blacks had a 9.0-fold increased risk (95% CI: 8.02-10.11; p<0.001) of injury due to assault than other intentions. Similarly, Hispanics were at the highest risk of assault (OR: 2.71; 95% CI: 2.363.12; p<0.001). Whites were more likely to suffer from self-inflicted injuries (OR: 10.53; 95% CI: 9.39-11.81; p<0.001) than other intentions (Figure 2B). The South was associated with the highest risk of FAOI compared with the other regions (OR: 1.29; 95% CI: 1.24-1.36; p<0.001). However, analysis based on racial/ethnic groups showed that Whites (OR: 1.20; 95% CI: 1.081.33; p=-0.001) and Hispanics (OR: 4.18; 95% CI: 3.67-4.75; p<0.001) were at the highest risk of FAOI in the West and Blacks in the Mid-West (OR: 1.69; 95% CI: 1.53-1.88; p<0.001) than other regions.

### Role of intention

Intention was found to be associated with the types of ocular injury, injury severity, and levels of TBI. Open globe (OR: 1.88; 95% CI: 1.52-2.17; p<0.001) and open adnexal wound injuries (OR: 1.72; 95% CI: 1.47-2.03; p<0.001) exhibited the highest likelihood of occurring in unintentional firearm injuries. Of note, other non-visual pathway cranial nerve injuries (OR: 1.78; 95% CI: 1.47-2.16; p<0.001) and open adnexal wounds (OR: 1.43; 95% CI: 1.29-1.5; p<0.001) were most likely to occur after assault injuries. Self-inflicted firearm injury was most associated with orbital injuries (OR: 1.79; 95% CI: 1.63-1.96; p<0.001), and optic nerve and visual pathway injuries (OR: 1.82; 95% CI: 1.56-2.14; p<0.001).

**Figure 3:**
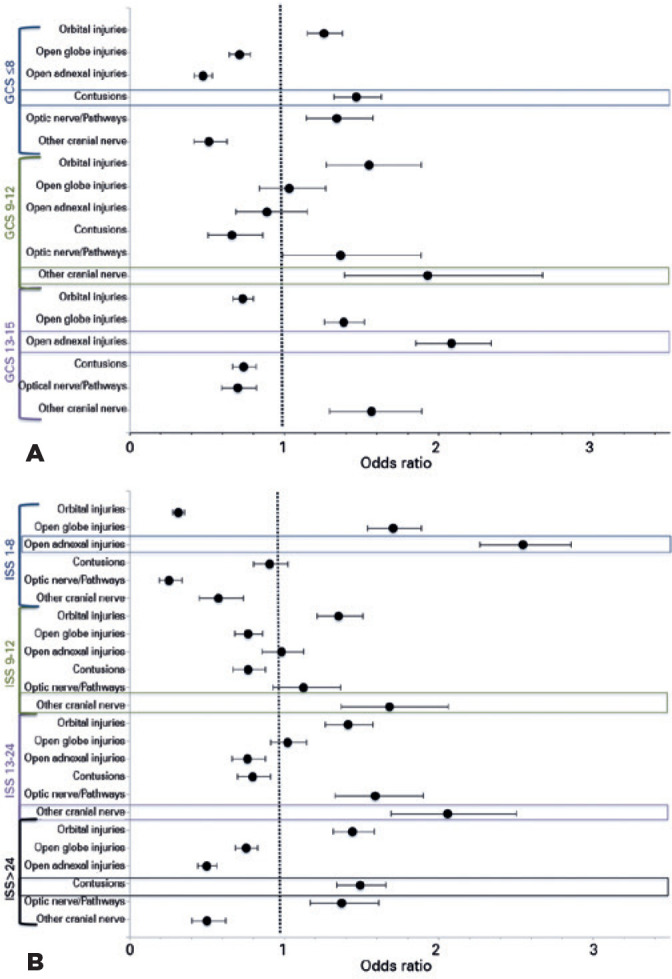
A) Simple logistic regression of ocular injuries and Glasgow Coma Scale in firearm-associated ocular injuries, NTDB (2008-2014). Summary of simple logistic regression with odds ratio and 95% confidence intervals analysis of injury type and injury severity (GCS) in firearmassociated ocular injury. Contusions (OR: 1.63, p<0.001), optic nerve and pathway (OR: 1.57; p<0.001), and orbital injuries (OR: 1.26; p<0.001) were associated with GCS scores <8 (severe TBI). Other cranial nerve (OR: 1.93; p<0.001) and orbital (OR: 1.55; p<0.001) were associated with GCS scores of 9-12 (moderate TBI). Open adnexal (OR: 2.08; p<0.001), open globe (OR:1.39; p<0.001), and other cranial nerve injuries (OR: 1.56; p<0.001) were associated with GCS scores of 13-15 (mild TBI). Boxed plots represent categories with the highest odds ratio. B) Simple logistic regression of ocular injuries and injury severity score in firearm-associated ocular injuries, NTDB (2008-2014). Summary of simple logistic regression with odds ratio and 95% confidence intervals analysis of injury type and injury severity (ISS) in firearm-associated ocular injury. Open adnexal (OR: 2.55; p<0.001) and open globe injuries (OR: 1.71; p<0.001) were associated with ISS (1-8: minor); other cranial nerve (OR: 1.68; p<0.001) and orbital (OR: 1.35; p<0.001) of ISS (9-12: moderate). Optic nerve and pathway (OR: 1.59; p<0.001) and orbital injuries (OR: 1.41, p<0.001) were associated with ISS (16-24: severe); contusions (OR: 1.49; p<0.001), orbital (OR: 1.44; p<0.001), and optic nerve and pathways of ISS >24, or the most severe injuries. Boxed plots represent categories with the highest odds ratio.

TBI (64.6%) was most likely to occur following self-inflicted FAOI (OR: 5.37; 95% CI: 4.74-6.08; p<0.001). Figures 3A and 3B illustrate the relative associations of ocular injuries with GCS (TBI) and ISS. Severe FAOI associated with low GCS (<8) (OR: 22.91; 95% CI: 18.85-27.84; p<0.001) and high ISS (>24) (OR: 14.88; 95% CI: 12.73-17.38; p<0.001) were associated with the highest risk of mortality. When analyzed based on intention, unintentional firearm injuries were linked to minor TBI (OR: 2.40; 95% CI: 2.03-2.83; p<0.001) and ISS (OR: 3.73; 95% CI: 3.21-4.33; p<0.001), while self-inflicted injuries were associated with severe TBI (OR: 5.34; 95% CI: 4.82-5.93; p<0.001) and very severe ISS (OR: 2.99; 95% CI: 2.71-3.29; p<0.001). Consequently, self-inflicted injuries were linked to the highest risk of mortality (OR: 3.60; CI: 3.20-4.04; p<0.001). Assault injuries were most associated with a risk of mild or intermediate injury severity compared with the other intentions; GCS: 13-15 (OR:3.16, 95% CI: 2.88-3.46; p<0.001) and ISS: 9-15 (OR: 1.70, 95% CI: 1.52-1.90; p<0.001).

NTDB= National Trauma Data Bank; GCS= Glasgow Coma Scale; OR= odds ratio; TBI= traumatic brain injury; ISS= Injury Severity Score; OR= odds ratio.

## DISCUSSION

The current dearth of literature addressing firearm injuries is incommensurate with the gravity of this public health issue in the USA. Ophthalmologists manage these patients frequently as patients with major trauma, admitted with multiple injuries. This study evaluated a national database to determine the scope of ophthalmic injuries incurred following firearm-associated injuries. Although we affirmed the common finding of trauma occurring most frequently in young males, additional findings revealed that FAOI were more strongly associated with older age groups (>40 years) and self-inflicted injury in Whites, while Blacks and Hispanics tended to be younger and victims of assault (Figures 2A and 2B). Furthermore, Blacks were disproportionately affected overall. Blacks represent only 13% of the USA population^(14)^ but account for 35% of all FAOI victims. Our study confirms the conclusions from other firearm trauma reports in the USA indicating that this demographic group represents an at-risk sub-population^(5,15)^. Intention of injury was also associated with different types of firearm-related injuries. Open globe and adnexal wound injuries were mostly associated with unintentional trauma, other non-visual pathway cranial nerve injuries were associated with assault and optic nerve/ visual pathway, and orbital injuries were associated with self-inflicted injury. Self-inflicted injury exhibited the strongest association with TBI and mortality.

Most reports of FAOI have concentrated on non-powder firearm injuries from air, paintball, pellet, and nail guns.^(16,17)^ with very few reports including handguns, shotguns, and rifles^(6,9)^. McGwin et al. investigated the epidemiology of both air gun (BB, pellet, paint, and rifles) and firearm (all powder guns) trauma using the National Electronic Injury Surveillance System (NEISS) and reported similar demographic findings to those of our study^(6)^. They found that young males were at the highest risk of injury; Blacks were more likely to be injured by firearms and assault, while Whites were more likely to be injured by air guns and unintentional injury. Fowler et al. utilized the National Vital Statistics and NEISS to describe fatal and nonfatal firearm-associated injuries in the USA, and reported similar demographic patterns^(15)^. Notably, they also identified intention as a predictor of injuries; homicides were more frequent in adolescents and young adults, while firearm suicide tended to increase with age. Their findings are consistent with those observed in this study. In a small regional analysis of two New York City hospitals, Chopra et al.^(9)^ also found similar patterns, with 3% of firearm injuries involving the eyes; assault and self-inflicted were the most common (64%) and least common (7%) intentions, respectively. They recorded a mean (SD) ISS and GCS score of 14.15 (9.69) and 12.85 (4.16), respectively. Although the rate of assault noted in the present study was comparable (56.8%), we observed a higher rate of suicide injuries (30.1%), which may account for the greater severity of injuries reported in our study. These differences highlight difficulties in comparing data from one setting with data from a large, inclusive national trauma database. Despite the differences, there appears to be consensus between studies identifying young, male, and minority populations as particularly vulnerable to firearm-associated injury^(6,7,13,15)^. Understanding these demographic patterns is crucial for identifying those who are most likely to benefit from future, targeted prevention programs.

In a retrospective, multicenter study, Shackford et al. investigated the association between firearm-associated injuries to the face and morbidity/mortality^(12)^. As expected, there was a high mortality rate, with 97% of deaths associated with brain injury. Ocular sequelae were frequent complications in those with non-fatal injuries. We found that more than a third of patients had GCS and ISS scores consistent with severe brain injury and very severe injury. Specifically, orbital injury, contusions, and optic nerve injuries were associated with higher severity scores. We also observed a high incidence of brain injury; nearly 65% of patients admitted for FAOI suffered TBI. All degrees of TBI can lead to short and long-term disruption of visual processing that includes, but is not limited to, abnormal saccades and smooth pursuit, convergence insufficiency, diplopia, and accommodative dysfunction^(18,19)^.

In a study of the United States Eye Injury Registry, Kuhn et al.^(20)^ found that 6% of all severe eye traumas were caused by firearms. Furthermore, they found that 28.5% of patients sustained bilateral injuries in firearm-associated injuries and 58% of eyes with FAOI remained blind after 6 months. Although our data did not include ophthalmic clinical details and visual outcomes, we know that most patients survived their injuries albeit with high rates of TBI. This suggests that survivors may experience complicated rehabilitation when considering the neurocognitive consequences of TBI. With this knowledge, healthcare providers managing patients with FAOI should consider coordinating ophthalmic and neurologic care, during a long-term follow-up beyond the post-operative period, to optimize visual outcomes^(21-23)^.

Our study identified self-inflicted injury or suicide as a major cause of FAOI in Whites and older adults. This intention also exhibited the strongest association with TBI, greater ISS, and mortality than other intensions. In an analysis of a World Health Organization mortality database, Richardson et al. found that 80% of all firearm-related deaths occurred in the USA. Despite having a 30% lower suicide rate than other high-income countries, the USA had a 5.8-fold higher rate of firearm-associated suicide^(1)^. Through the Center for Disease Control questionnaire data (2000-2002), Miller et al. found that with every 1% increase in firearm ownership, the rate of firearm-related suicide increased by 3.5%^(24)^. They concluded that since firearms are implicated in >50% of suicides in the USA, reductions in firearm ownership would drastically reduce the rate of firearm-related suicide. With the progressive aging of the USA population, there exists a growing need to develop strategies for identifying these high-risk elderly patients^(1,25,26)^. Although effective worthy treatment algorithms have been developed to restore appearance and functionality in patients maxillofacial and ocular injuries after failed suicide attempts^(27)^, associated residual visual compromise and the high rate of TBI and mortality in elderly individuals warrants the creation of targeted prevention strategies.

In a recent study investigating pediatric FAOI, Weiss et al.^(28)^ found that this group represented 22.6% of all cases, with 62.9%, 17.5%, and 13.1% caused by assault, unintentional injury, and self-inflicted injury, respectively. Most injuries (38.6%) occurred at home. When considering preventive strategies in the pediatric group, Barkin et al.^(29)^ found that >40% of homes had ≥1 unlocked firearm and deduced that >1.7 million children aged <18 years are living in homes in which loaded and unlocked firearms are present. Through a randomized controlled trial, they showed that office-based violence-prevention discussions led to increased firearm storage and decreased child media usage, factors that they suggest contribute to firearm-associated injuries. The recent establishment of hospital-based violence intervention programs to target high-risk, injured patients, has been found to be cost-effective and reduce recidivism. These programs aim at reducing obstacles to services and improve behavior that decreases exposure to violence^(30)^. We identified assault and self-harm injuries as major intentions at both ends of the age spectrum that led to the most severe injuries and an increased susceptibility to TBI. Ophthalmologists could play an important role in the initial surgical intervention and subsequent TBI management, as well as in reducing the incidence of future firearm-related injuries by engaging patients in similar violence prevention strategies or by appropriately referring them to available services.

The main limitation of this study was its retrospective design. Although extensive, NTDB data were not submitted by ophthalmologists, but rather by trauma/ emergency room personnel who may have underestimated ophthalmic injuries. Also, ophthalmic clinical details and long-term visual outcomes were not available. ICD9-CM codes were used during this period and do not describe injuries with the same degree of accuracy as ICD-10-CM codes. Furthermore, we evaluated patients admitted with major trauma, which likely skewed the data towards more severely injured patients. However, given the severity of firearm-associated injuries and the wide reach of the NTDB, the general patterns elucidated herein may provide valuable insight and a sound foundation for further investigation.

In conclusion, we found that FAOI were often sight-threatening and associated with TBI. Intentions were associated with age groups, gender, race and ethnicity as well as injury severity and the degree of TBI. Further ICD10-CM NTDB analysis needs to be conducted to confirm the present findings and enhance our knowledge in this field.
